# Retinal phototoxicity and the evaluation of the blue light hazard of a new solid-state lighting technology

**DOI:** 10.1038/s41598-020-63442-5

**Published:** 2020-04-21

**Authors:** Imene Jaadane, Gloria Villalpando Rodriguez, Pierre Boulenguez, Samuel Carré, Irene Dassieni, Cecile Lebon, Sabine Chahory, Francine Behar-Cohen, Christophe Martinsons, Alicia Torriglia

**Affiliations:** 1 INSERM U1138, Centre de Recherches des Cordeliers, Université Paris Descartes, Université Pierre et Marie Curie, Paris, France; 2 CSTB, Centre Scientifique et Technique du Bâtiment, Division Eclairage et électromagnétisme, Saint Martin d’Heres, France; 3 ENVA, Ecole Nationale Vétérinaire d’Alfort, Unité d’ophtalmologie, Maisons-Alfort, France

**Keywords:** Retina, Risk factors

## Abstract

Exposure Limit Values (ELV) for artificial lighting were defined in order to prevent light-induced damage to the retina. The evaluation of the lighting devices include the correction of their spectra by the B(λ) function or blue light hazard function, representing the relative spectral sensitivity of the human eye to the blue light. This weighting function peaks between 435 and 440 nm. In this study we evaluate a new generation of light emitting diode (LED), the GaN-on-GaN (gallium nitride on gallium nitride) LED, that present an emission peak in the purple part of the spectrum. Wistar rats were exposed to GaN-on-GaN and conventional diodes at different retinal doses (from 2.2 to 0.5 J/cm^2^). We show that GaN-on-GaN diodes are more toxic than conventional LED for the rat neural retina and the rat retinal pigment epithelium, indicating that the BLH (blue light hazard) weighting is not adapted to this type of diodes. One of the reasons of this increased toxicity is the effects of shorter wavelengths on mitochondria polarization. We also show that the threshold of phototoxic retinal dose in the rat (fixed at 11 J/cm^2^, BLH weighted) is overestimated, suggesting that the values used for regulations, calculated in primates using the same methods than in rats, should be revised.

## Introduction

Exposure Limit Values (ELV), proposed by the ICNIRP (International Commission for Non-Ionizing Radiation Protection) were defined in order to prevent light-induced photochemical damage to the retina (blue light hazard). These limits were used in the EN NF 62471 standard that define four groups of photobiological risk for incoherent (non laser) light sources ranging from risk group 0, concerning light sources delivering a retinal dose up to 2.2 J/cm^2^ in 10 000 s, that are thought to be no risk, to risk group 3 for which an exposure of 0.25 s or less might be harmful for the retina. It is worth noticing that the retinal dose corresponds to the amount of blue light reaching the retina. Actually, the spectra of the measured lighting devices is corrected using the B(λ) function. The B(λ) function, also called the blue light hazard function represents the relative spectral sensitivity of the human eye to the blue light hazard. It is based upon the relative spectral effectiveness of optical radiation to induce retinal photochemical injury (photic maculopathy)^1,2^. This weighting function peaks near 445 nm and has a profile close to the sensitivity of short-wave cones. The attenuation of sensitivity for shorter wavelength visible light (<440 nm) is caused by the absorption of the lens of the eye and the cornea^3^.

Most of currently used LED are Gallium Nitride-based (GaN) grown on top of sapphire or silicon substrate. In the last few years a new LED technology was developed using GaN substrates, generating GaN-on-GaN diodes (gallium nitride on gallium nitride)^4^. The use of the GaN substrate greatly improves the light emission. These diodes can be operated at higher current densities and produce more light from a smaller area. Their short wavelength emission is shifted to the purple part of the spectrum (around 405 nm) and they use a mix of three phosphors giving a better color rendering while avoiding the blue overshoot and the cyan gap of conventional LED.

Classically described, two types of photochemical damages are induced by light: the first involves rhodopsin and affects photoreceptors^5,6^. The second concerns the RPE (retinal pigment epithelium), selectively vulnerable to high-energy blue photons^7^. We have previously shown that conventional LED (light emitting diode) light cause retinal injury in rats. This includes the activation of apoptosis and necrosis^8,9^. The blue component of light emitted by the LED is the major cause of this damage. Moreover, the blue component of the white-LED may cause retinal toxicity at occupational household illuminance levels and not only in extreme experimental conditions^10^. We have also shown^9^ that after LED exposure at doses below the recognized toxic levels, there is a permeabilization of the outer blood retinal barrier (OBRB), a feature seen in many common retinopathies (diabetic retinopathy, for instance)^11^ together with an increase of the cells size, both features related to ageing of the RPE cells^12^.

In this paper we investigate the effects of conventional LED and GaN-on-GaN LED on rat retina using different doses and at different times after exposure. We also investigate the possible biological mechanisms involved in the difference of sensitivity found between the two generations of LED. The relevance of using the BLH (blue light hazard) weighting in assessing the risk group of household lighting devices is also discussed.

## Results

### **Damage is induced at a total dose of 2.2 J/cm**^2^

**Figure 1:**
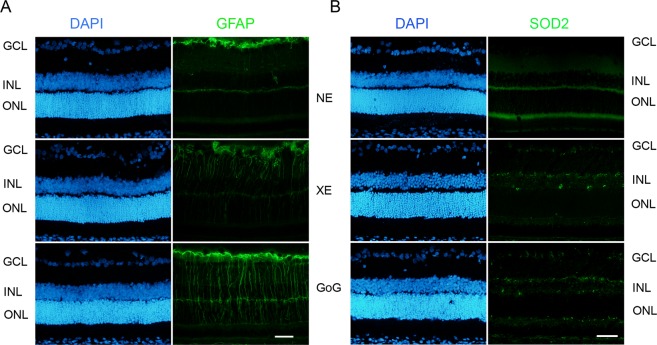
Oxidative stress response after a total dose of 2.2 J/cm^2^. Male Wistar rats aged 7 weeks were exposed to GaN-on-GaN (GoG) LED or conventional white LED (XE) (Xanlite XXX Evolution 5 W) for 9 and 10 h respectively at 1900 lx, receiving the estimated retinal dose of 2.2 J/cm^2^ (BLH weighted 0.253 J/cm^2^ for regular LED and to 0.26 J/cm^2^ for GaN-on-GaN LED). 15 hours after the end of the exposure period, the eyes were included in optimal cutting temperature medium (Tissue Tek), cryosectioned and immunostained. NE: Non exposed. (**A**) sections were stained with anti-Glial Fibrillary Acid Protein (GFAP) and (**B**) with anti-superoxide dismutase 2 (SOD2). Nuclei were stained in blue with DAPI. ONL indicates the outer nuclear layer, INL the inner nuclear layer and GCL the ganglion cell layer. Scale bar represents 100 µm. Photographs show representative images of the upper retina.

**Figure 2:**
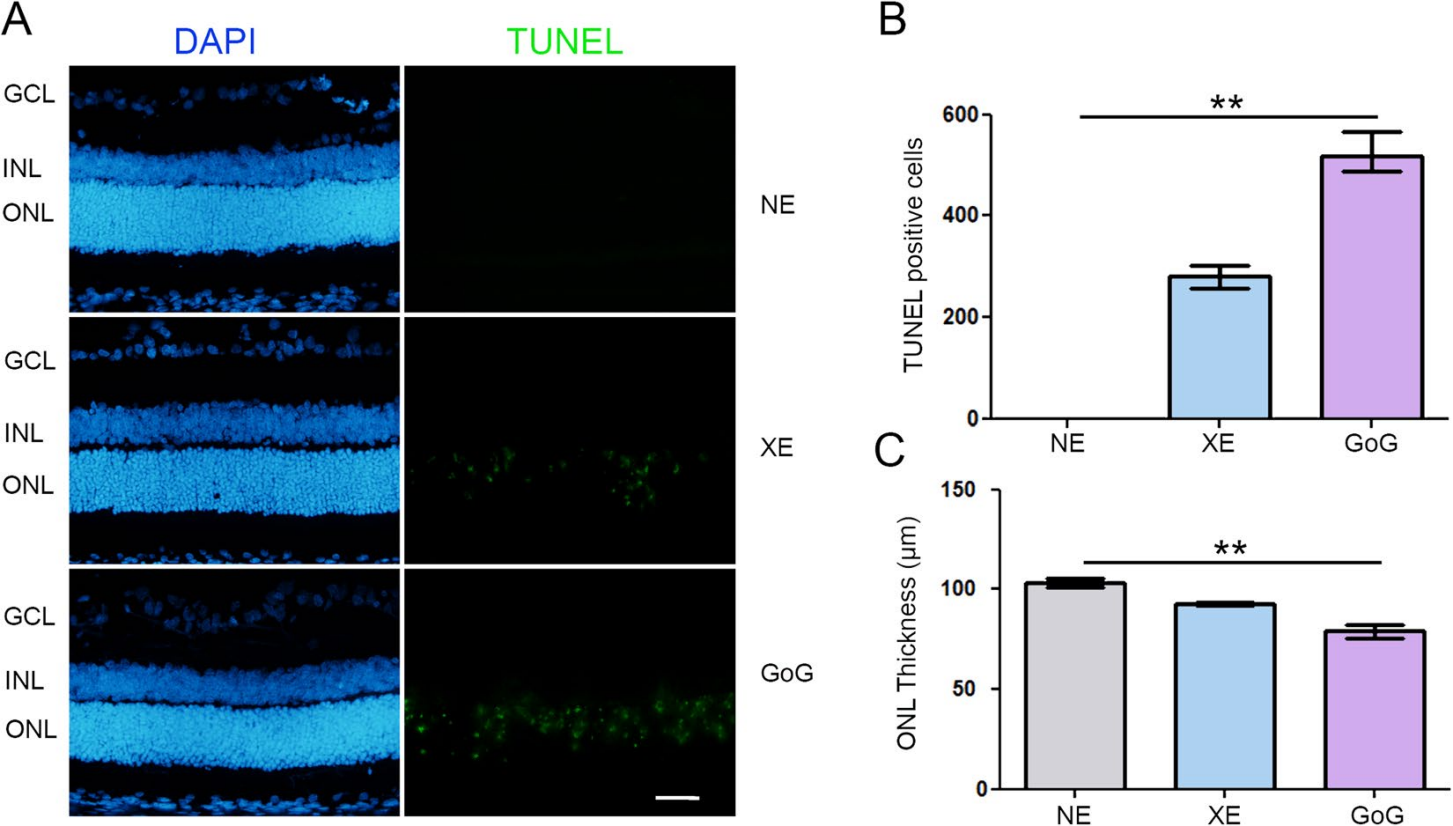
Photoreceptors cell death after a total dose of 2.2 J/cm^2^. Male Wistar rats aged 7 weeks were exposed to GaN-on-GaN (GoG) LED or conventional white LED (XE) (Xanlite XXX Evolution 5 W) for 9 and 10 h respectively at 1900 lx, receiving the estimated retinal dose of 2.2 J/cm^2^ (BLH weighted 0.253 J/cm^2^ for regular LED and to 0.26 J/cm^2^ for GaN-on-GaN LED). 15 hours after the end of the exposure period the eyes were included in optimal cutting temperature medium (Tissue Tek), cryosectioned and immunostained. NE: Non exposed. (**A**) 15 hours after the end of the exposure period the eyes were included in optimal cutting temperature medium (Tissue Tek), cryosectioned and stained with the TUNEL assay (TUNEL). Nuclei were stained in blue with DAPI. ONL indicates the outer nuclear layer, INL the Inner nuclear layer and GCL the ganglion cell layer. Scale bar represents 100 µm. Photographs show representative images of the upper retina. (**B**) Quantification of TUNEL positive cells in exposed retinas from independent experiments. Histograms represent the median with the interquartile range. Significance was evaluated using the Kruskal-Wallis test followed by the Dunn’s multiple comparison post-test. H(3) = 10.83 044. **p ≤ 0.1, n = 4. (**C**) Quantification of the thickness of photoreceptors nuclei layer in exposed retinas. Histograms represent the median with the interquartile range. Significance was evaluated using the Kruskal-Wallis test followed by the Dunn’s multiple comparison post-test. H(3) = 9.846. **p ≤ 0.05, n = 4.

In order to perform a first comparison between GaN-on-GaN and conventional white LED we used a retinal dose of 2.2 J/cm^2^. This dose was obtained exposing the animals at 1900 lx for 9 hours for GaN-on-GaN LED and 10 h for Xanlite Evolution. The difference in the exposure time is due to technical constraints. This corresponds to a global dose of 2.2 J/cm^2^ and to a dose at 10 000 s (normative framework) of 0.68 J/cm^2^. At the neural retinal level we found an increase of stress markers like glial fibrilary acidic protein (GFAP) (Fig. 1A), and a decrease of antioxidant enzymes like superoxide dismutase 2 (SOD2) at the photoreceptor level (Fig. 1B). These effects are more important for GaN-on-GaN than for regular (Xanlite evolution, XE) LED. Moreover, we found an induction of cell death with an increased amount of TUNEL (Terminal transferase dUTP nick end labeling) positive cells in GaN-on-GaN exposed animals as compared to conventional LED (Fig. 2A,B). As this labeling indicates the amount of cell death at the moment at which the experiment was done and could not be representative of the total amount of cell death, we evaluated the remaining photoreceptors left after the experiment by counting the number of nuclei of the outer nuclear layer (ONL) one week after light exposure (Fig. 2C). A decreased number of nuclei was found in retinas subject to LED light but the decrease was more important with GaN-on-GaN diodes (Fig. 2C).

### **A global dose of 0.5 J/cm**^2^**is still toxic for the retina**

**Figure 3:**
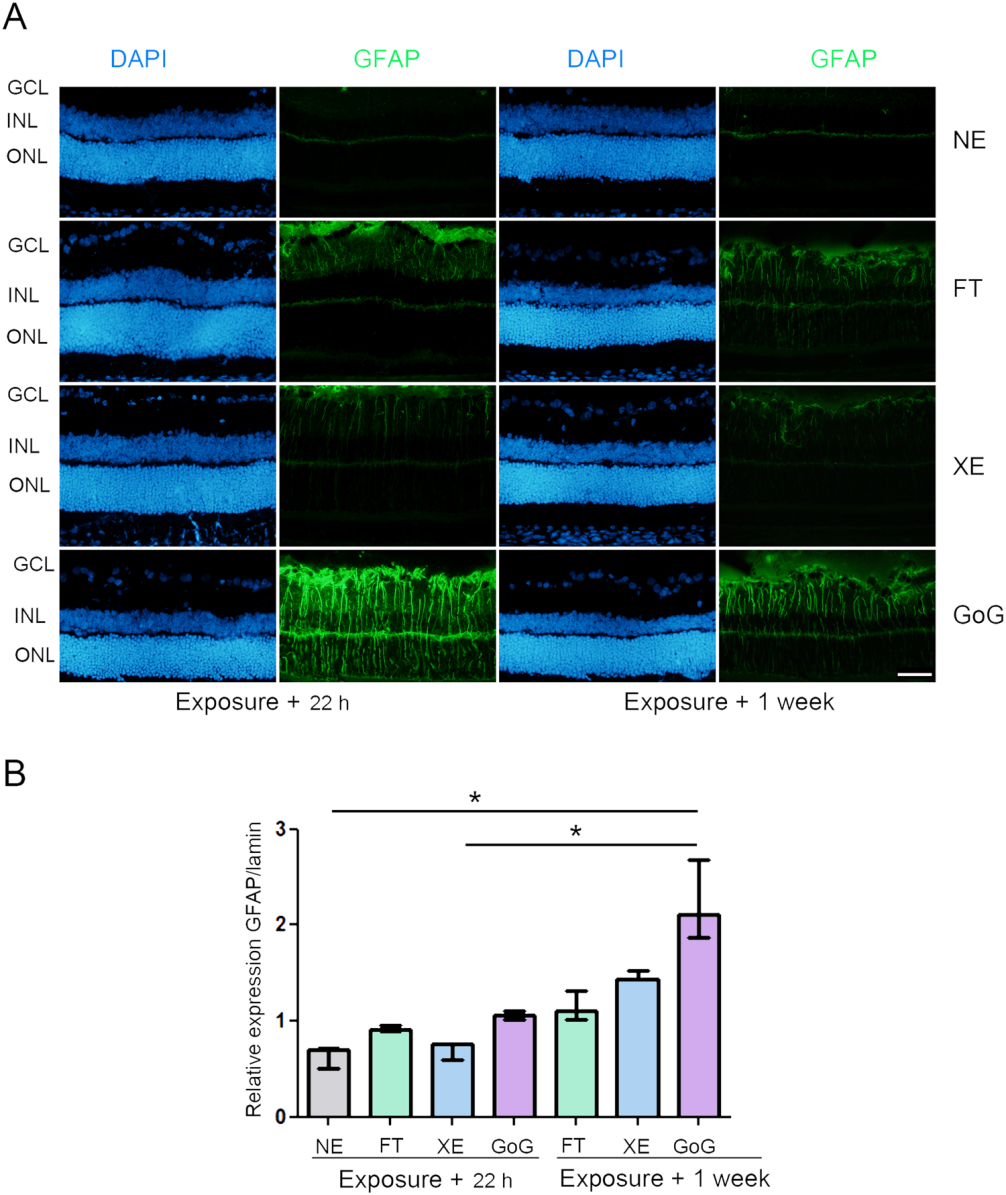
GFAP stress response after a total dose of 0.5 J/cm^2^. Male Wistar rats aged 7 weeks were exposed to GaN-on-GaN (GoG) LED or conventional white LED(XE) (Xanlite XXX Evolution 5 W) or fluorescent tubes (FT) at 1900 lx, receiving the estimated retinal dose of 0.5 J/cm^2^ (2 h for GoG, 2h20 min for XE and FT). 22 hours or 1 week after the end of the exposure period (**A**) the eyes were included in optimal cutting temperature medium (Tissue Tek), cryosectioned and immunostained with anti-Glial Fibrillary Acid Protein (GFAP) (green). NE: Non exposed. Nuclei were stained in blue with DAPI. ONL indicates the outer nuclear layer, INL the inner nuclear layer and GCL the ganglion cell layer. Scale bar represents 100 µm. The presented photographs were taken in the superior part of the retina, at 200 µm from the optic nerve. (**B**) the eyes were enucleated, the retina dissected, extracted with M-PER buffer and loaded on the top of a 10% SDS-PAGE, transferred onto a nitrocellulose membrane and probed with anti-GFAP. Lamin B was used as a charge control. Histograms represent the median with the interquartile range. Significance was evaluated using the Kruskal-Wallis test followed by the Dunn’s multiple comparison post-test. H(7) = 19.09. *p ≤ 0.1, n = 3.

**Figure 4:**
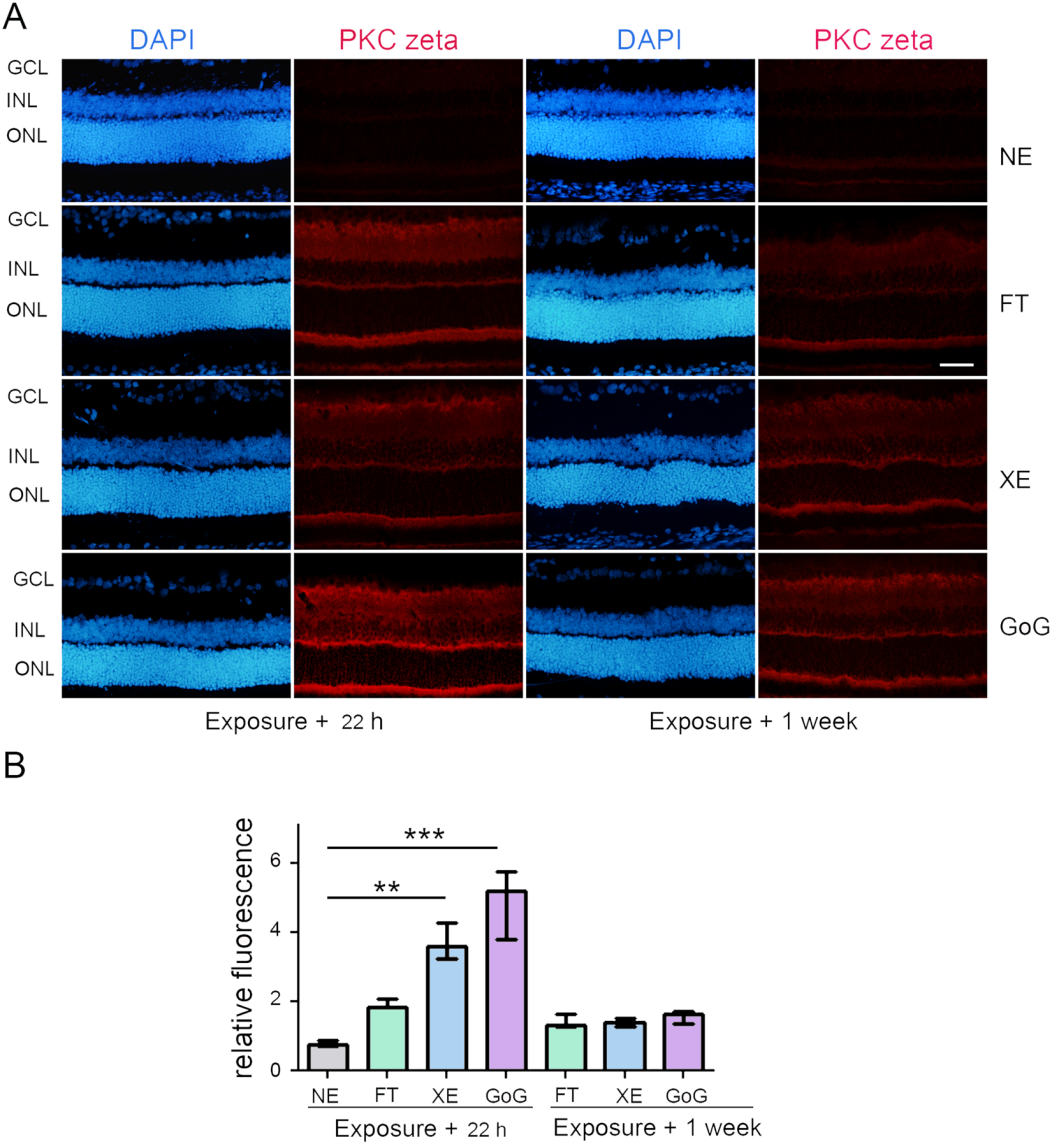
PKC zeta stress response after a total dose of 0.5 J/cm^2^. Male Wistar rats aged 7 weeks were exposed to GaN-on-GaN (GoG) LED or conventional white LED (XE) (Xanlite XXX Evolution 5 W) or fluorescent tubes (FT) at 1900 lx, receiving the estimated retinal dose of 0.5 J/cm^2^ (2 h for GoG, 2h20 min for XE and FT). 22 hours or 1 week after the end of the exposure period. (**A**) 22 hours or 1 week after the end of the exposure period the eyes were included in optimal cutting temperature medium (Tissue Tek), cryosectioned and immunostained with anti-protein kinase C zeta (red). NE: Non exposed. Nuclei were stained in blue with DAPI. ONL indicates the outer nuclear layer, INL the inner nuclear layer and GCL the ganglion cell layer. Scale bar represents 100 µm. The presented photographs were taken in the superior part of the retina, at 200 µm from the optic nerve. (**B**) Quantification of the relative fluorescence of PKC zeta. Histograms represent the median with the interquartile range. Significance was evaluated using the Kruskal-Wallis test followed by the Dunn’s multiple comparison post-test. H(7) = 45.80.**p ≤ 0.05, n = 4.

The global dose of 2.2 J/cm^2^ being toxic for the two devices investigated here, we explored lower doses by simply using the same protocol and decreasing the time of exposure from 9 h to 2 h. This decreased the global dose from 2.2 to 0.5 J/cm^2^. In this set of experiments we added a third source of light: fluorescent tubes used at the same exposure parameters. This was done in order to have a point of comparison because most of the phototoxicity data found in the literature used fluorescent tubes. The retina was evaluated 22 h after the exposure and a week later in order to see the reversibility or not of the damages produced. Figure 3 shows the results concerning GFAP and Fig. 4 PKC zeta (protein kinase C zeta). In all cases there is a strong reaction of the tissue that decreases 1 week after exposure. It is worth noticing that the GaN-on-GaN device presented the strongest and the more persistent reaction.

**Figure 5:**
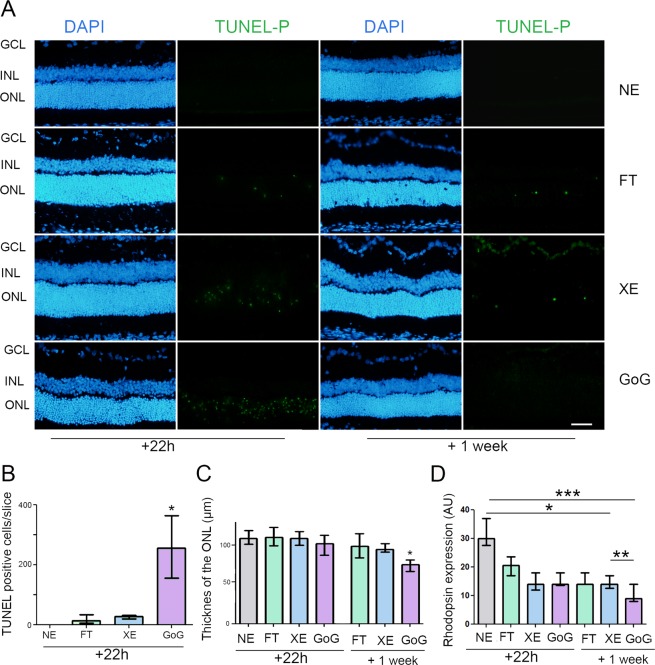
Photoreceptors cell death after a total dose of 0.5 J/cm^2^. Male Wistar rats aged 7 weeks were exposed to GaN-on-GaN (GoG) LED or conventional white LED (XE) (Xanlite XXX Evolution 5 W) or fluorescent tubes (FT) at 1900 lx, receiving the estimated retinal dose of 0.5 J/cm^2^ (2 h for GoG, 2h20 min for XE and FT). 22 hours or 1 week after the end of the exposure period. (**A**) 22 hours or 1 week after the end of the exposure period the eyes were included in optimal cutting temperature medium (Tissue Tek), cryosectioned and stained with the TUNEL assay (TUNEL) with a previous dephosphorylation step. Nuclei were stained in blue with DAPI. NE: Non exposed. ONL indicates the outer nuclear layer, INL the Inner nuclear layer and GCL the ganglion cell layer. Scale bar represents 100 µm. (**B**) Quantification of TUNEL positive cells in exposed retinas 22 hours after illumination. Histograms represent the median with the interquartile range. Significance was evaluated using the Kruskal-Wallis test followed by the Dunn’s multiple comparison post-test. H(4) = 14.57. **p ≤ 0.05, n = 4. (**C**) Quantification of the number of photoreceptors nuclei in exposed retinas. Histograms represent the median with the interquartile range. Significance was evaluated using the Kruskal-Wallis test followed by the Dunn’s multiple comparison post-test. H(7) = 18.72. *p ≤ 0.05, n = 4. (**D**) quantification of rhodopsin expression. Histograms represent the median with the interquartile range. Significance was evaluated using the Kruskal-Wallis test followed by the Dunn’s multiple comparison post-test. H(7) = 25.33. *p ≤ 0.05, n = 4.

Although the global dose was about 0.5 J/cm^2^, we still had a high amount of cell death as indicated by the TUNEL labeling (Fig. 5A). When the labeling was made 22 hours after GaN-on-GaN LED exposure we saw the highest amount of cell death (Fig. 5B). The evaluation of the remaining photoreceptors one week after exposure showed that only under GaN-on-GaN LED exposure we had a significant decrease of the remaining photoreceptors (Fig. 5C). Note that some cells were still dying a week later. This was confirmed by the measure of the rhodopsin expression (Fig. 5D).

### Is the illuminance level of GaN-on-GaN LED involved in retinal damage?

**Figure 6:**
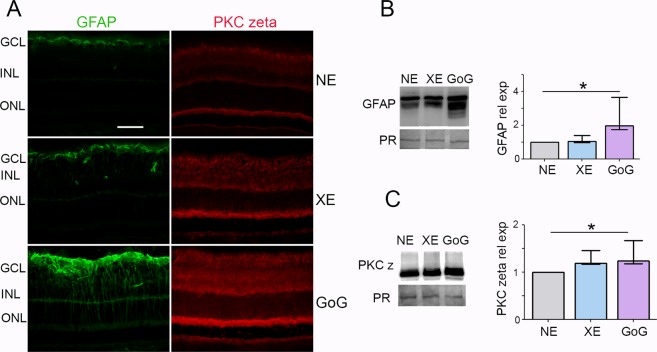
Stress response after a total dose of 1 J/cm^2^. Male Wistar rats aged 7 weeks were exposed to GaN-on-GaN (GoG) LED for 2 hours at 4000 lx or conventional white LED (XE) (Xanlite XXX Evolution 5 W) for 1 h 10 min at 8000 lx, receiving the estimated retinal dose of 1 J/cm^2^. (**A**) 24 hours after the beginning of the exposure period the eyes were included in optimal cutting temperature medium (Tissue Tek), cryosectioned and immunostained with anti-Glial Fibrillary Acid Protein (GFAP)(green) and with anti-protein kinase C zeta (PKC zeta) (red). ONL indicates the outer nuclear layer, INL the Inner nuclear layer and GCL the ganglion cell layer. NE: Non exposed. Scale bar represents 100 µm. The presented photographs were taken in the superior part of the retina, at 200 µm from the optic nerve. (**B**) the eyes were enucleated, the retina dissected, extracted with M-PER buffer and loaded on the top of a 10% SDS-PAGE, transferred onto a nitrocellulose membrane and probed for GFAP. PR indicates Ponceau Red staining (PR). Histogram shows the quantification of the western blot, they represent the median with the interquartil range. Significance was evaluated using the Kruskal-Wallis test followed by the Dunn’s multiple comparison post-test. H(3) = 8.29. *p ≤ 0.05, n = 4. (**C**) same experiment that in panel B using anti-PKC zeta H(3) = 7.930. *p ≤ 0.05, n = 4.

**Figure 7:**
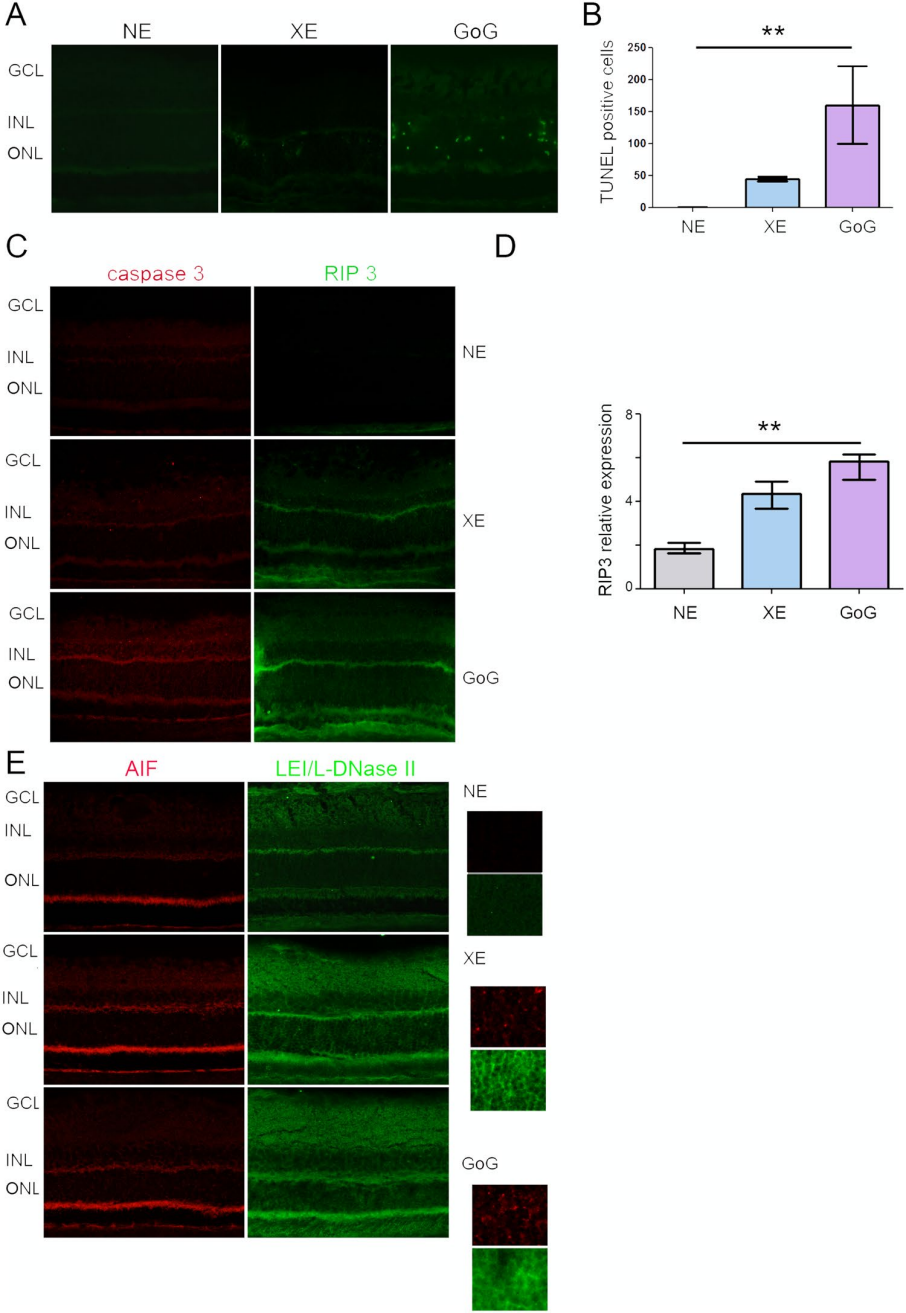
Photoreceptors cell death after a total retinal dose of 1 J/cm^2^. Male Wistar rats aged 7 weeks were exposed to GaN-on-GaN (GoG) LEDfor 2 hours at 4000 lx or conventional white LED (XE) (Xanlite XXX Evolution 5 W) for 1 h 10 min at 8000 lx, receiving the estimated retinal dose of 1 J/cm^2^. (**A**) 24 hours after the beginning of the exposure period the eyes were included in optimal cutting temperature medium (Tissue Tek), cryosectioned and stained with the TUNEL assay (TUNEL)(green). Nuclei were stained in blue with DAPI. ONL indicates the outer nuclear layer, INL the Inner nuclear layer and GCL the ganglion cell layer. NE: Non exposed. Scale bar represents 100 µm. (**B**) Quantification of TUNEL positive cells in exposed retinas. Histograms represent the median with the interquartile range. Significance was evaluated using the Kruskal-Wallis test followed by the Dunn’s multiple comparison post-test. H(3) = 12.59. **p ≤ 0.05, n = 4. (**C**) same experiment that in panel A using anti-active caspase 3 (red) or anti- Receptor interacting protein 3 kinase (RIP3) (green). ONL indicates the outer nuclear layer, INL the Inner nuclear layer and GCL the ganglion cell layer. Scale bar represents 100 µm. (**D**) Quantification of the relative fluorescence of RIP 3 kinase. Histograms represent the median with the interquartile range. Significance was evaluated using the Kruskal-Wallis test followed by the Dunn’s multiple comparison post-test. H(3) = 12.20. **p ≤ 0.05, n = 5. (**E**) same experiment that in panel A using anti-apoptosis interacting factor (AIF) (red) or anti-leukocyte elastase inhibitor/L-DNase II (LEI/L-DNase II) (green).

In the previous experiments we used the same photometric exposure and the equivalent exposure time to compare the effects of different lighting devices. The retinal dose depends on the illuminance level and on the exposure time for a given device, when all other conditions are kept unchanged. According to the equation of Sliney and Van Norren (see Materials and Methods), it also depends on the spectrum of the source. In order to give some insight into the biological weight of these different parameters we performed experiments in which the retinal dose was the same for classical LED and GaN-on-GaN LED diodes but different illuminance levels and exposure times were chosen. As the results presented in the previous section showed that for the same exposure conditions GaN-on-GaN LED were more toxic to the rat retina than conventional LED, we chose to expose our animals to a more intense illuminance level of conventional LED. The resulting total retinal dose was of 1 J/cm^2^ but conventional LED were used at an illuminance level of 8000 lx and GaN-on-GaN LED were used at 4000 lx. Figure 6A shows immunolabelling of the exposed retinas in which the tissue stress was seen through an increase of GFAP and PKC zeta labeling. This increase was also evaluated by western blot and quantified (Fig. 6B). With no surprise regarding the previous section (because we are at a retinal dose of 1 J/cm^2^), retinal stress (revealed by an increased expression of GFAP and PKC zeta, is induced (Fig. 7A), but it is stronger with GaN-on-GaN LED than with classical LED (Fig. 7B). The investigation of cell death effectors (Fig. 7C) shows an activation of the L-DNase II, a caspase independent cell death effector which is translocated to the nucleus upon activation (see magnification of Fig. 7E) and an increased RIP (receptor interacting protein) expression, a necroptosis marker (Fig. 7D).

### The retinal pigment epithelium (RPE)

**Figure 8:**
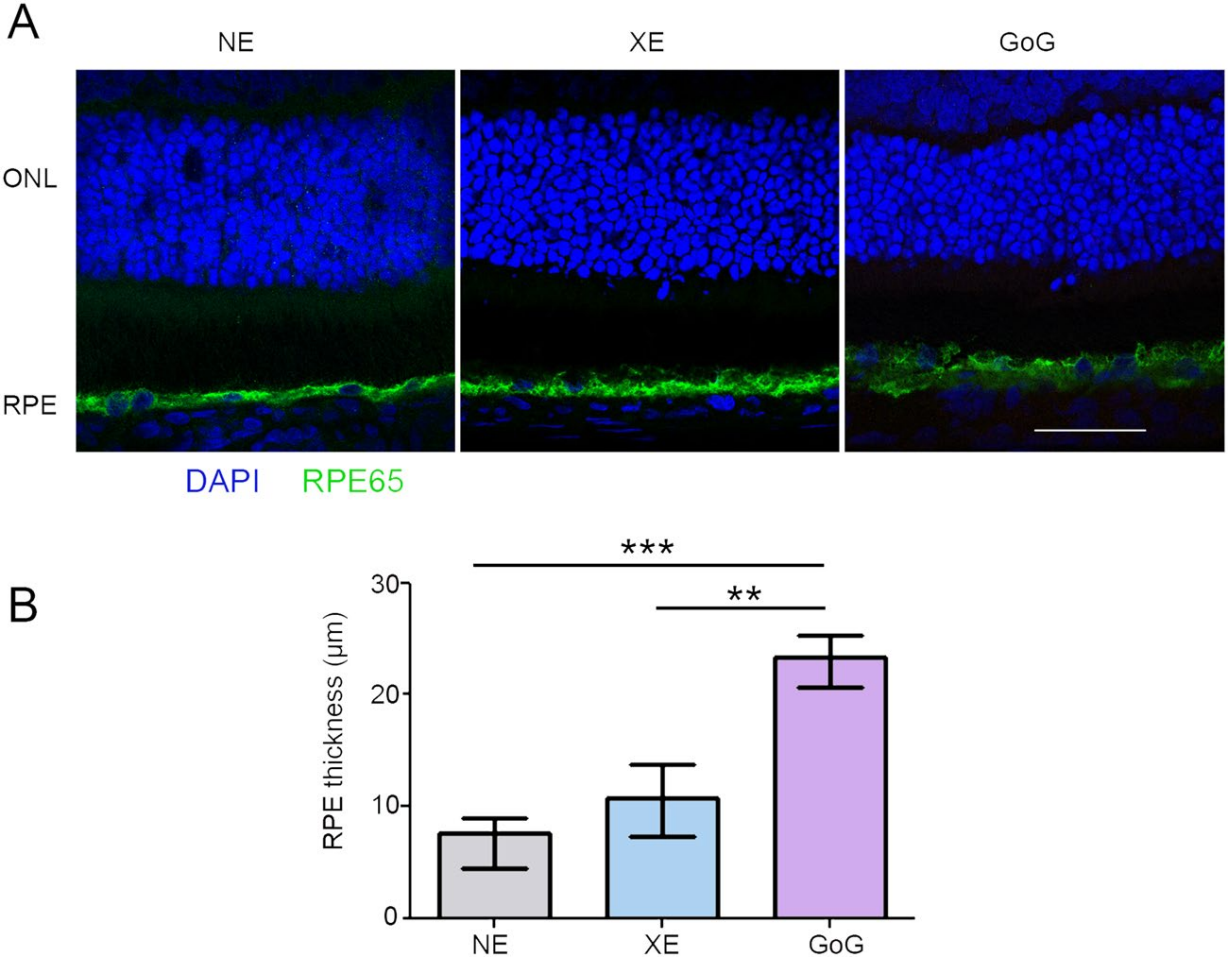
RPE damages induced by a total retinal dose of 2.2 J/cm^2^. Male Wistar rats aged 7 weeks were exposed to GaN-on-GaN (GoG) LED or conventional white LED (XE) (Xanlite XXX Evolution 5 W) for 9 and 10 h repectively at 1900 lx, receiving the estimated retinal dose of 2.2 J/cm^2^ (BLH weighted 0.253 J/cm^2^ for regular LED and to 0.26 J/cm^2^ for GaN-on-GaN LED). (**A**) 15 hours after the end of the exposure period the eyes were included in optimal cutting temperature medium (Tissue Tek), cryosectioned and immunostained with anti-retinal pigment epithelium protein 65 (RPE 65). Nuclei are stained with DAPI in blue. ONL indicates the outer nuclear layer, RPE the retinal pigment epithelium. Scale bar represents 100 µm.NE: Non Exposed. (**B**) Measurement of the RPE thickness after exposure. Histograms represent the median with the interquartile range. Significance was evaluated using the Kruskal-Wallis test followed by the Dunn’s multiple comparison post-test. H(3) = 26.28. ***p ≤ 0.001, n = 12.

The experiments described above indicated that the toxic thresholds levels of light currently used are overestimated. However, the use of rats in our experiments raised many questions regarding the transposition of these results to human owing to the difference in retina structures and phototoxicity thresholds. We investigated then the effects of this exposure on RPE, a retinal tissue more similar between species. At 2.2 J/cm^2^ we found an increase in the thickness of the RPE (Fig. 8).

**Figure 9:**
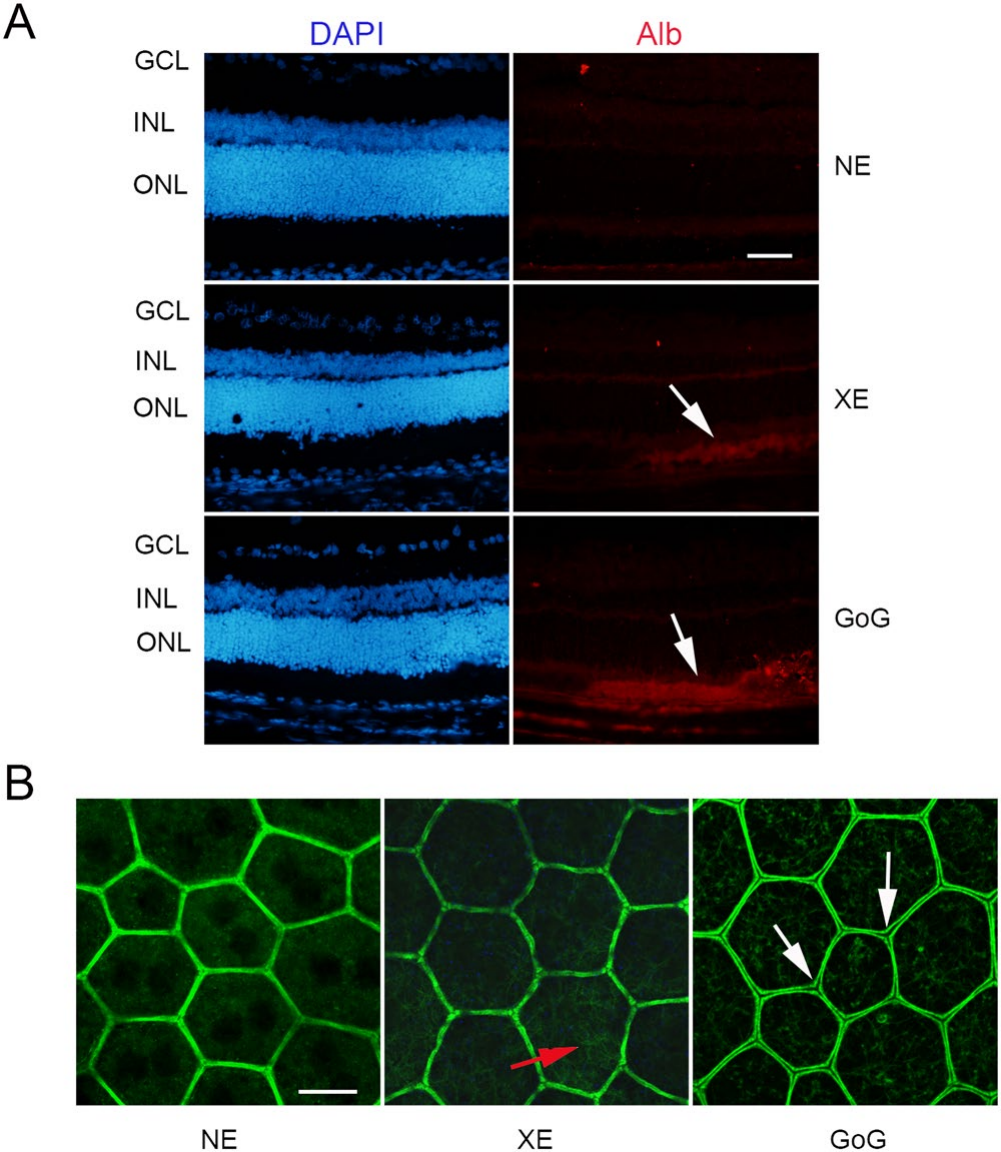
The RPE after a total retinal dose of 2.2 J/cm^2^. Male Wistar rats aged 7 weeks were exposed to GaN-on-GaN (GoG) LED or conventional white LED (XE) (Xanlite XXX Evolution 5 W) for 9 and 10 h respectively at 1900 lx, receiving the estimated retinal dose of 2.2 J/cm^2^ (BLH weighted 0.253 J/cm^2^ for regular LED and to 0.26 J/cm^2^ for GaN-on-GaN LED). (**A**) 15 hours after the end of the exposure period the eyes were included in optimal cutting temperature medium (Tissue Tek), cryosectioned and immunostained with anti-Rat seric albumin (alb). White arrows indicate albumin leakage. ONL indicates the outer nuclear layer, INL the inner nuclear layer and GCL the ganglion cell layer. NE: Non exposed. Scale bar represents 100 µm. (**B**) alternatively the eyes were dissected and the posterior pole containing the RPE and the choroid was flat mounted, stained with labeled phalloidin (green) and analyzed under a confocal microscopy. Red arrow indicates stress fibers, white arrows show interruptions of the OBRB. Scale bar represents 10 µm.

**Figure 10:**
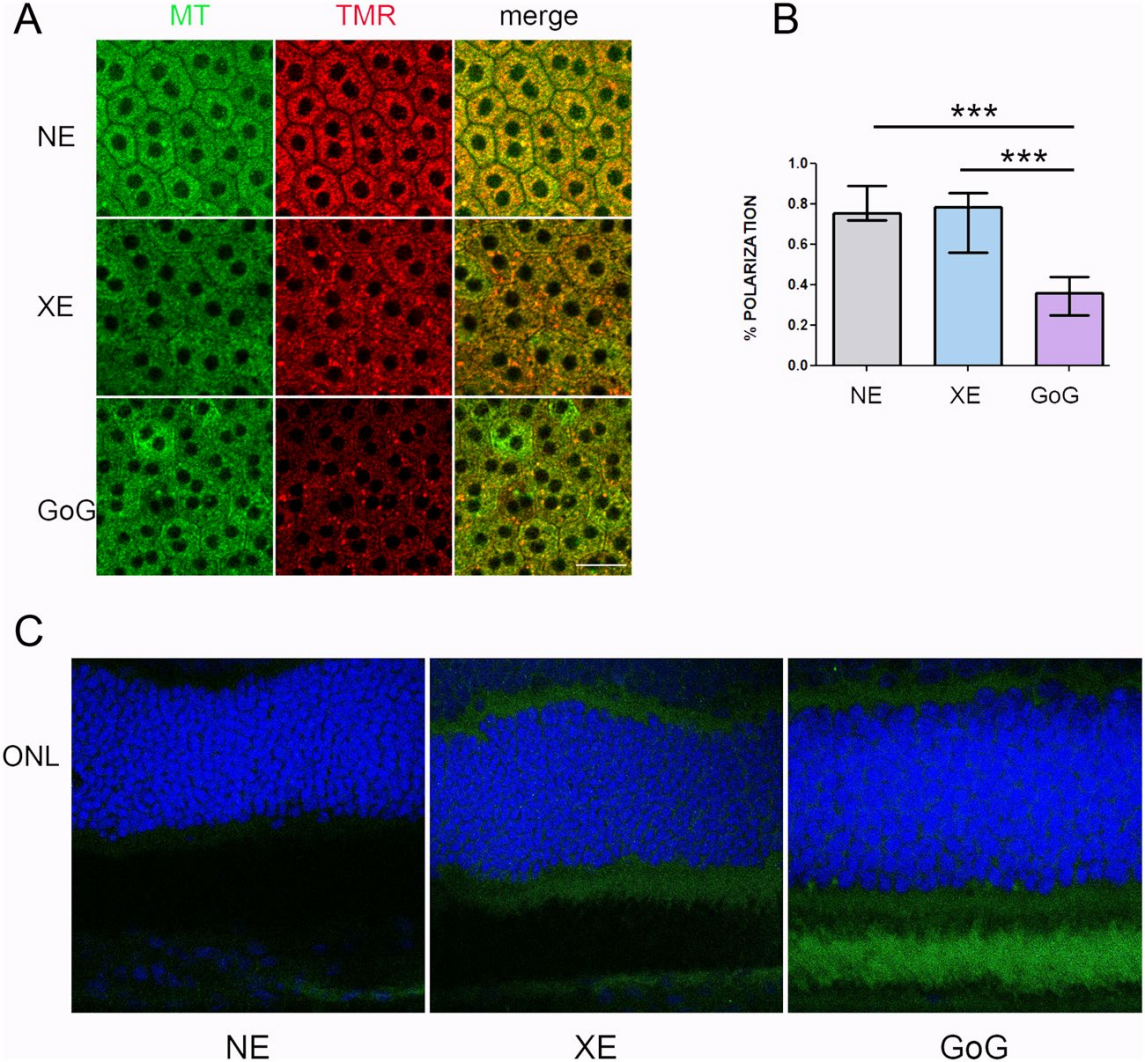
The RPE mitochondria after a total dose of 2.2 J/cm^2^. Male Wistar rats aged 7 weeks were exposed to GaN-on-GaN (GoG) LED or conventional white LED (XE) (Xanlite XXX Evolution 5 W) for 9 and 10 h respectively at 1900 lx, receiving the estimated retinal dose of 2.2 J/cm^2^ (BLH weighted 0.253 J/cm^2^ for regular LED and to 0.26 J/cm^2^ for GaN-on-GaN LED). (**A**) 15 hours after the end of the exposure RPE was flat mounted and mitochondria were labeled with mitotracker green (MR) and with thrimethylrhodamine (TMR). Scale bar represents 20 µM. NE: Non exposed. (**B**) by calculating the rate of colocalization of MR and TMR the rate of polarized mitochondria was calculated. Histograms represent the median with the interquartile range. Significance was evaluated using the Kruskal-Wallis test followed by the Dunn’s multiple comparison post-test. H(3) = 45.17. ***p ≤ 0.001, n = 11. (**C**) 15 hours after the end of the exposure period the eyes were included in optimal cutting temperature medium (Tissue Tek), cryosectioned and stained in blue with anti-RAGE (green). Nuclei are stained with DAPI. Scale bar represents 100 µm.

Some localized leakage of the outer retinal barrier was found when exposed to classical LED, but an important leakage was seen after GaN-on-GaN LED exposure (Fig. 9A). This correlated with a faint opening of the outer retinal barrier, as seen on Fig. 9B. In order to have some insight in the difference of sensitivity to GaN-on-GaN diodes we looked for the absorbance spectra of different components of the retina, seeking for a component able to absorb around 410 nm. We found that the flavins are able to absorb in this range of wavelength^13^. Flavins are major co-factors of enzymes belonging to the Krebs’ cycle and to the respiratory chains. As these enzymes are mostly located in the mitochondria we studied the membrane potential of RPE mitochondria (Fig. 10). This was done by colabelling mitochondria with the potential sensitive Tetramethyl rodamine and the mitochondrial marker mitotracker green (Fig. 10A). The quantification showed a slight non significant increase in the number of depolarized mitochondria in RPE coming from a regular LED exposed retina, while a significant decrease was measured in GaN-on-GaN LED exposed tissues (Fig. 10B). Interestingly, the labeling of retinal slices with an anti RAGE (receptor for advanced glycation end products) shows an increased labeling in RPE of GaN-on-GaN exposed animals (Fig. 10C).

## Discussion

In this study we show, using different retinal doses and exposure protocols, that GaN-on-GaN light emitting diodes are more toxic that conventional diodes for the neural retina and the retinal pigment epithelium. We also show that one of the reasons of this increased toxicity might be the effects of shorter wavelengths on mitochondria. Finally, we show that the threshold of phototoxic retinal dose in the rat (11 J/cm^2^, BLH weighed) is largely overestimated.

As mentioned in the introduction, the GaN-on-GaN LED present an emission peak in the purple part of the spectrum avoiding the blue overshoot and the cyan gap of conventional LED. For this reason, it was said that they were safer for the retina, because of the absence of the blue peak. Actually, if we compare the GaN-on-GaN LED and the conventional LED (Xanlite evolution) used in this study, their BLH is equivalent when using the same corneal irradiance. However, we have shown here that GaN-on-GaN LED are more harmful for the retina in all the tested conditions and for all the markers tested. This is also true for the RPE. Moreover, our results comparing same retinal doses with higher classical LED irradiance still show a higher toxicity of GaN-on-GaN LED. These results strongly suggest the involvement of the used light spectrum in this phenomenon. Moreover, these results suggest that the BLH weighting, although appropriate for regular LED, is not adapted for other devices emitting shorter wavelength light.

The current regulations for household and professional lighting classify light sources, lamps and luminaires according to their emission characteristics. Lamps classified in risk group 0 are defined as providing less than 2.2 J/cm^2^ of BLH weighed retinal dose in 10 000 s when placed at a distance of 20 cm from the eye^14^. The value of 2.2 J/cm^2^ was determined from primates phototoxicity curves^2^: the primate threshold of phototoxicity for wavelength between 400–450 nm was experimentally fixed at 22 J/cm^2^. This threshold was reduced by a safety margin of 10 to establish the exposure limit values. In the same paper, Van Norren *et al*. compare primate and rodent curves. The threshold in the same wavelength range is 11 J/cm^2^ for rodents, highlighting the higher sensitivity to light of these animals. It is important to note that the methods used to calculate those values were the same for primates and rodents. In this paper we show that at a total irradiance dose of 0.5 J/cm^2^ we already have cell death. Moreover, if we consider the BLH weighting, this global dose corresponds to 0.06 J/cm^2^. This means that for rats we are detecting cell death with a dose of blue light about 180 fold smaller than the exposure limit values. As we said before, the same readout was done in rodents and primates. These results suggest that the phototoxicity data obtained in primates, and used to establish the current regulations must also be re-evaluated.

Although neural retina in humans and rodents have differences in organization and sensitivity to light the RPE is highly analogous, it has the same functions and ages in the same way in most of the species^15^. Therefore, phototoxicity data from animal models concerning RPE should be more closely related to phototoxicity in humans. Actually, the accumulation of lipofuscin, for instance, is a major event in humans and in animals during RPE ageing^16^. Here we show that the exposure to light emitted by GaN-on-GaN LED affects RPE mitochondria in a higher extent than classical LED, although an effect is also seen with the latter. Mitochondria being the major organelle of energy production, it is highly understandable that its depolarization might contribute to the decreased selectivity of the OHRB seen after GaN-on-GaN LED exposure, explaining the leakage of albumin seen in these exposure conditions. It is worth noting that the decreased selectivity of the OBRB and the leak of choroid fluid into the interphotoreceptor space is characteristic of RPE in AMD (Age related macular degeneration) and in diabetic retinopathy. In addition, the high extent of mitochondria depolarization might create an overload of the autophagolysosomal system leading to an accumulation of lipofuscin. This pigment is photosensibilizing and it also impairs phagocytosis, one of the most important functions of the RPE^17^. Interestingly an increase of RAGE expression is seen. The expression of this receptor is induced in inflammatory settings and is often seen in diabetes and diabetic retinopathy where it plays an important role in sustaining inflammation^18^.

In conclusion, we show here that the use of the standard BLH function in order to provide photobiological risk group of lamps and luminaries might not be stringent enough. Devices like GaN-on-GaN LED can have the same BLH risk group than regular LED but be more harmful for the retina. We also show that in rats the photosensitivity threshold has been largely overestimated. As the same methods were used to estimate photosensitivity in primates and the limit exposure values, a re-evaluation of these values should be considered. Finally, the effects of light emitted by GaN-on GaN LED on RPE present features reminding the modifications seen in AMD, raising the question of a possible link between the exposure to short wavelength light and the increase of the incidence of AMD.

## Methods

### Animals

Three-week-old male Wistar rats were purchased from Charles Rivers labs Rivers and left in the animal facilities for 3 weeks for acclimation in a light cycle of 12 h (about 100 lx during the lighting period). All procedures were performed according to the animal use and care committee of the National Veterinary School of Alfort (referenced as n°2016031712195939 were approved by the ethical committee of ENVA).

### Light source

Two types of lighting devices built and characterized by the Lighting and Electromagnetism division of the Scientific and Technical Center for Building (CSTB, Saint Martin d’Heres, France) were used. One contains only white LED (Xanlite XXX Evolution 5 W), while the other device is a half sphere supporting GaN-on-GaN bulbs commercially available and manufactured from Soraa Inc. Details from the device and the emission spectra are shown on additional Fig. 1. The exposure doses were calculated with a dedicated software developed by the CSTB for our experiments (Photobiology Dosimeter). Calculations relied on *in situ* spectrophotometric measurements and on a model of the Wistar rat vision and behavior. The retinal doses were calculated according to Sliney 1984^19^ and Van Norren 2011^2^.

$${\rm{H}}={\rm{pt}}\frac{\pi {\rm{d}}2}{4{{\rm{f}}}^{2}}{\int }_{0}^{\infty }{\tau }_{\lambda }{{\rm{L}}}_{\lambda }{\rm{d}}\lambda [{\rm{J}}/{\rm{cm2}}].$$

${\rm{p}}$ = posture coefficient, ${\rm{t}}$ = exposure time, ${\rm{d}}$ = diameter of the pupil, ${\rm{f}}$ = focal length, τ_λ_ = spectral transmittance of the rat ocular media, L_λi_ = spectral radiance of the source. We chose p = 0.9, and according to Van Norren *et al*. was chosen at 0.9^2^, (d = 5 mm, f = 5.25 mm). Although the pupil is not dilated, we choose 5 mm because we used albino rats and presumed then that their iris did not absorb much light.

According to a discussion with Pr. van Norren, we estimated the τλ value by associating the rat spectral transmittance proposed by Gorgels^20^ with the human cornea transmission published by Van den Berg^21^.

### Light exposure

Wistar rats were kept in transparent cages placed under the light sources, they were exposed to different retinal doses of light as indicated in the different figures without been previously dark-adapted or pupil dilated (in the rat is maximal at about 300 cd/m^2^). Exposure started at 9AM, so that the longest exposures were kept into the subjective day of the animal facility.

### Tissue preparation

Rats were sacrificed with a lethal intraperitoneal injection of Sodium pentobarbital (Ceva, La Ballastiere, France). Eyes were enucleated, embedded in optical cutting temperature (OCT) compound (Tissue Tek compound Sakura) and stored at −80 °C. 10 μm cryosections were cut using a cryostat (Leica CM 3050 S) and stored at −20 °C. For biochemical experiments, neuroretina was frozen immediately at −20 C. Preparation of RPE was done as in Jaadane *et al*.^9^.

For some experiments, the RPE-choroid complex was kept in Minimum Essential Medium (MEM) and used to evaluate mitochondrial potential.

### Western blot

Protein extraction from light exposed retinas was performed as described in Jaadane *et al*.(8). Protein concentration was measured with the BCA Protein Assay Kit (Thermo Scientific, Illkirch, France), according to the manufacturer’s instructions. Proteins were diluted in Laemmli sample buffer, separated in a 12% SDS–PAGE, immobilized on nitrocellulose membrane (Protran, Whatman, GE Healthcare, Versailles, France) and blotted with specific primary antibody at 1/1000 dilution: anti-PKC zeta (Santa Cruz sc-216, Clinisciences, Nanterre, France), anti-phospho-PKC zeta (Santa Cruz sc-12894R, Clinisciences, Nanterre, France), anti-Actin (Santa Cruz sc-1616, Clinisciences, Nanterre, France), anti-GFAP (Dako, Z 0334) and anti-Receptor Interacting Protein (RIP3 Sigma-Aldrich PRS2283). The secondary antibodies horse radish peroxydase (HRP)-conjugated (Vector, Eurobio, Les Ulis, France) were used at 1/5000 dilution. Luminata Forte Western HRP Substrate (Millipore, Merck Chimie, Fontenay sous Bois, France) was used to reveal the signal.

### Immunohistochemistry

Immunolabelling were performed as described in Jaadane *et al*.^9^. Briefly, cryosections were washed with PBS (phosphate buffered saline, Life Technology) containing Ca^2+^ and Mg^2+^, fixed in 4% paraformaldehyde for 15 min at room temperature and then washed with PBS. Permeabilization was performed with 0.3% Triton X-100 for 20 min, blocked by 1 h incubation in 1% non-fat milk in PBS and incubated with specific primary antibodies in 0.1% non-fat milk in PBS diluted at 1/100 for 1 h. The primary antibodies used were against Glial Fibrilary acidic protein: (GFAP, Aligent-Z033429), Superoxyde dismutase 2 (SOD2, R&D Systems), protein kinase C zeta (PKC zeta Thermo Fisher 38–1400), active caspase 3 (Cell Signaling 9661), receptor interacting protein 1;(RIP, BD Laboratories 610469), rat albumin (Thermo Fisher PA1–29266), retinal pigment epithelium 65 (RPE 65, Thermo Scientific 401.8B11.3D9). This was followed by incubation for 1 h with a 1/500 dilution of specific secondary antibodies (Invitrogen, Life Technologies, Saint Aubin, France), 5 min with 4,6 di-aminidino-2-phenyl indoledichloride (DAPI) (1/5000), (Sigma Aldrich, Saint-Quentin Fallavier, France) and coverslipped with fluoromount (Sigma, Saint-Quentin Fallavier, France). Immunoreactivity was visualised using fluorescence microscopy with an Olympus microscope BX51 (Olympus France, Rungis, France) or a Zeiss LSM 710 confocal microscope.

### Terminal transferase dUTP nick end labeling (TUNEL)

Cryosections were fixed in 4% paraformaldehyde 15 min and permeabilized with 0.3% Triton X-100 in PBS for 20 min. After washes, the TUNEL assay was performed according to the manufacturer’s instructions (Roche Molecular Biochemical’s, Meylan, France). The sections were then rinsed with PBS, incubated with DAPI 1/5000 for 5 min and coverslipped with fluoromount (Sigma Aldrich, Saint-Quentin Fallavier, France). Immunoreactivity was visualized as before.

### Mitochondrial depolarization in RPE

After dissection, RPE choroid complexes were kept in MEM containing 1% Fetal Calf serum at 37 °C in a 5% CO_2_ humidified oven. The mitochondria were stained with both mitotracker green (Cell signaling) (binding cardiolipin and staining all the mitochondria) and with tetramethyl rhodamine methyl ester (TMRM) (retained only in polarized mitochondria). The staining was performed by incubation 20 min at 37 °C in red phenol free DMEM containing 20 mM Hepes, 50 nM TMRM and and 200 nM Mitotracker green.The samples were then mounted in PBS containing Calcium Magnesium and 10 mM glucose in a thermoregulated chamber, humidified and containing 5% CO2, of a Zeiss confocal microscope.

### Ethics

All procedures were in compliance with the animal use and care committee of the National Veterinary School of Alfort.

## Supplementary information

Additional Information

## Data Availability

The datasets used and/or analysed during the current study are available from the corresponding author on reasonable request.
