# Supplementary material for: Retinal phototoxicity and the evaluation of the blue light hazard of a new solid-state lighting technology
Source: Sci Rep. 2020 Apr 21;10:6733. doi: 10.1038/s41598-020-63442-5 (PMC7174369; doi:10.1038/s41598-020-63442-5)
Supplement: Supplementary file 1 — Additional Information [file 41598_2020_63442_MOESM1_ESM.pdf]

ADDITIONAL FIGURE 1 to: Retinal phototoxicity and the evaluation of the blue light hazard of a new solid-state lighting technology.

Imene Jaadane, Gloria Villalpando Rodriguez, Pierre Boulenguez, Samuel Carré, Irene Dassieni, Cecile Lebon,Sabine Chahory, Francine Behar-Cohen, Christophe Martinsons, Alicia Torriglia.

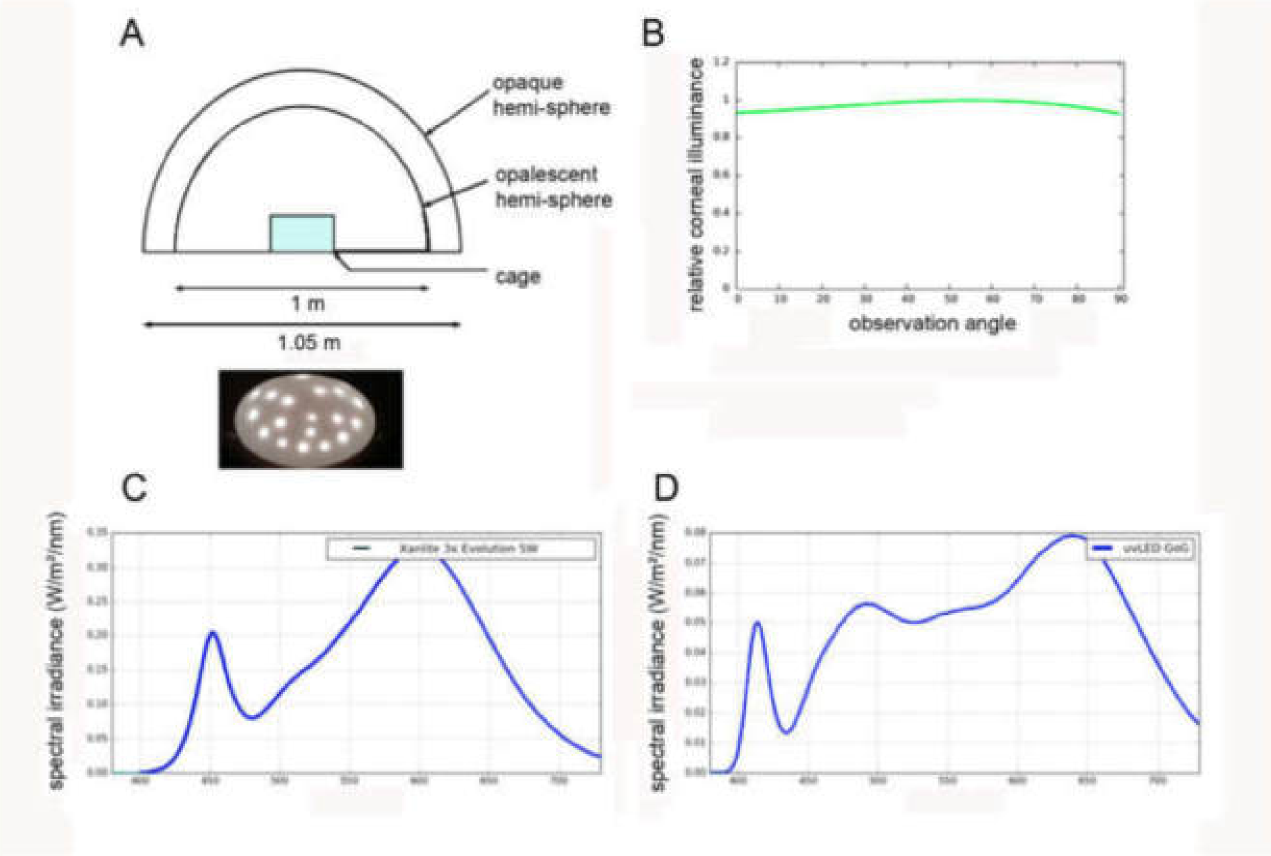

Spectral patterns of the LEDs used in this study.
A: The GaN-on-GaN LEDs (Soraa Inc.) were mounted in a half sphere with the indicated measurements. Underneath, the device in working conditions. B: Relative corneal illuminance with GaN-on-GaN LED as a function of the angle of the animal head. C: The emission spectrum of the Xanlite LEDs. D: The emission spectrum of the Soraa GaN-on-GaN LEDs.

Additional information:

Concerning the exposure device:

The exposure device was a diffuse hemispherical light source with a diameter of 1 m. It was built using two plastic half-spheres. An opaque outer half-sphere was used to support the light sources which were equally distributed across its surface. The inner half-sphere was made of opal polycarbonate, in order to diffuse and homogenize the emitted light. The resulting luminance uniformity was about 7% on the inner surface.

Layout of the LEDs on the outer opaque sphere. Each cross mark represents an individual light source.

The exposure device could be mechanically tilted to place the cage inside. The cage was placed at the center of the half-spheres.

The temperature inside the exposure device was controlled with a temperature sensor. A flow of air was continuously introduced inside to let the animals breathe and to avoid the build-up of excess heat in the device.

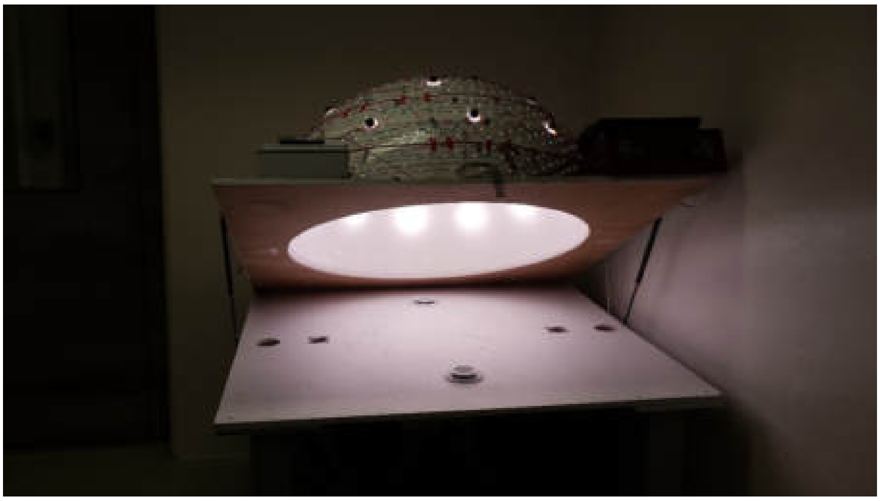

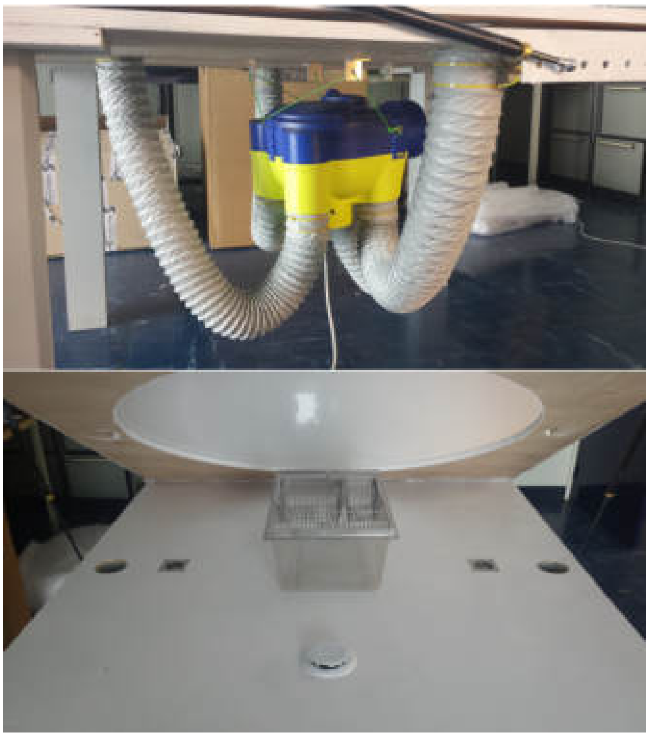

All the LEDs used in the device were previously seasoned for at least 100 h and installed after reaching a stable light output. The light levels and light spectra were measured at the location of the cage (center of the device). Before and after the experiments, the illuminance levels were measured to check the stability of the device.

The transmission spectrum of the cage was not measured. However, the light spectra and irradiance levels were measured using sensors placed within the transparent cage. As the cage was made of a neutral uncolored transparent material, it did not introduce any spectral distortion of the light emitted by the device.

Concerning the light doses used in this paper:

IEC 62471 is a safety standard for light sources. It defines a classification of light sources based on risk groups. The risk groups are defined by the exposure time required to exceed the internationally accepted limit values set by ICNIRP. By definition of IEC 62471, a light source classified in risk group 0 (no risk) does not exceed the ICNIRP exposure limit in 10 000s, at 20 cm from the source.

Since the IEC 62471 is a standard on light sources, it expresses the exposure limit in terms of light source quantities, not retinal quantities.

The latest ICNIRP recommendations are found in:

International Commission On Non-Ionizing Radiation Protection (Icnirp) Guidelines On Limits Of Exposure To Incoherent Visible And Infrared Radiation, Health Physics 105(1):74-96; 2013

For the retinal blue light hazard, the ICNIRP exposure limits are the following:

For exposure time between 0.25 s and 10 000 s :

Exposure limit DELB = 10 000 $J/m^2/sr$: this a dose of BLH-weighted radiance of light source

For exposure time exceeding 10 000 s :

Exposure limit LELB = 100 $W/m^2/sr$: this a BLH-weighted radiance of light source (not a dose anymore)

The ICNIRP guidelines give a formula to compute retinal irradiance as a function of source radiance (equation 2 in the guidelines). Using this formula allows us to express the exposure limit in terms of retinal irradiance dose. With the human eye parameters used in the guidelines (transmittance = 0.9, effective focal length = 17 mm, pupil diameter 3 mm), the exposure limit in terms of retinal irradiance dose is exactly 2.2 J/m^2 , for exposure times between 0.25 s and 10 000 s.

The retinal exposure limit of 2.2 J/cm^2 for blue light is also explicitly given as a "basic restriction" in another ICNRIP publication:

International Commission On Non-Ionizing Radiation Protection (ICNIRP) Adjustment Of Guidelines For Exposure Of The Eye To Optical Radiation From Ocular Instruments, Published In Applied Optics 44(11):2162-2176; 2005

The paper of Van Norren (reference 2, figure 1 right panel) recapitulates several studies in photo toxicology, and shows a photosensitivity threshold for blue light for the monkey of 22 J/cm^2 .This is higher by a factor of 10 compared with the ICNIRP basic restriction, which is a logical safety factor.

The threshold value for the monkey is 22 J/cm^2. But for the rat, this value is lower: 11 J/cm^2. This is not found in the ICNIRP guidelines but in the paper of van Norren (figure 1, left panel). If we impose to this value the same protection factor that was used for the monkey. This gives a complete "safe" dose of 1.1 J/cm^2. This was the reason for using 1 J/cm^2.

It is important to note that at 1 J/cm^2 the damage of the retina is still important. Therefore we made new experiments at 0.5 J/cm^2, half of the dose, to see if this dose is toxic.

<u>Concerning the BLH function:</u>

The BLH function (action spectrum) takes into account the Ham's damage to the retina, that is the so called type II damage. The type I damage described by Noell affects mostly photoreceptors and particularly rods. The use of the BLH function to evaluate phototoxicity in humans not considering type I damage comes from the fact that human retina is rich in cones and thus, only the damage of RPE (type II) seems relevant.

However, it has to be considered that the exclusive presence of cones in the retina only involves the macula. The rate of cones/rods decreases as we go from the macula to the periphery. In periphery, type I damage could be significant. This is important for vision: people presenting retinitis pigmentosa lose their peripheral vision and keeps only a tunnel vision, making their life very difficult. They are legally blind. In addition, the shrinking of the visual field is a known feature in human

ageing. We agree with the fact that our results are not transposable to human and even less to human macula but they can give some idea of the light toxicity in human peripheral retina.

Moreover, the use of the BLH discards the opposite part of the visual spectrum, this is the red part. This is very important since protective effects for different cells including retinal cells retina have been described in the literature. These effects are called photo-modulation. So that, it would be probably not the same thing to be exposed to a light with an balanced spectrum or to a light poor in red wavelength. This was discussed in the ANSES report of 2019 (https://www.anses.fr/fr/system/files/AP2014SA0253Ra.pdf). In addition, the use of BLH function assumes that wavelength below 425 nm are efficiently absorbed by the lens and the cornea. This is true for adults but not for children.

In conclusion, the BLH function is very useful to evaluate photochemical damage to the macula but it skips the effects induced by other wavelengths (green and red) that can have synergic or antagonic effects in retinal cells. The data that we show here shows this fact experimentally, using commercially available lamps.
